# Selective Feeding of a Mixotrophic Dinoflagellate (*Lepidodinium* sp.) in Response to Experimental Warming and Inorganic Nutrient Imbalance

**DOI:** 10.3389/fmicb.2022.805306

**Published:** 2022-04-19

**Authors:** Kailin Liu, Herrick Yin-To Ng, Zuyuan Gao, Hongbin Liu

**Affiliations:** ^1^Department of Ocean Science, The Hong Kong University of Science and Technology, Kowloon, Hong Kong SAR, China; ^2^Southern Marine Science and Engineering Guangdong Laboratory (Guangzhou), Guangzhou, China; ^3^State Key Laboratory of Marine Pollution, Hong Kong SAR, China

**Keywords:** mixotrophs, ingestion, warming, nutrient imbalance, prey quality

## Abstract

Mixotrophic protists are widely observed in the aquatic ecosystems, while how they respond to inorganic nutrient imbalance and ocean warming remains understudied. We conducted a series of experiments on a mixotrophic dinoflagellate *Lepidodinium* sp. isolated from subtropical coastal waters to investigate the combined effect of temperature and medium nitrate to phosphate ratio (N:P ratio) on the ingestion activities of mixotrophic protists. We found *Lepidodinium* sp. displayed selective feeding behaviour with a higher ingestion rate on high-N prey (N-rich *Rhodomonas salina*) when the ambient inorganic N:P ratio was equal to or below the Redfield ratio. The Chesson selectivity index α increased with increasing temperature, suggesting that warming exacerbated the selective feeding of *Lepidodinium* sp. Under inorganic nitrogen sufficient conditions (N:P ratio = 64), no selective feeding was observed at 25 and 28°C, while it occurs at 31°C, which also indicates that warming alters the feeding behaviour of *Lepidodinium* sp. In addition, our results revealed that the total ingestion rate of *Lepidodinium* sp. under the condition with normal inorganic nutrients (Redfield ratio) was significantly lower than that under nutrient-imbalanced conditions, which indicates that *Lepidodinium* sp. developed compensatory feeding to balance their cellular stoichiometry and satisfy their growth. Our study is the first attempt on revealing the selective feeding behaviours of mixotrophic protists on prey under different inorganic nutrient environments and rising temperatures, which will contribute to our understanding of the response of marine plankton food web to projected climate changes.

## Introduction

Mixotrophy is a nutrition strategy combining phototrophy and phagotrophy within one organism, ubiquitous among protists in aquatic ecosystems (Flynn and Mitra, 2009; Mitra et al., 2016). Phagotrophic algae, one common type of mixotrophs, are capable of acquiring carbon from photosynthesis as well as ingestion of prey (Stoecker, 1998; Mitra et al., 2016). Many studies have found that phagotrophic algae can benefit from prey ingestion, which allows them to thrive in the waters where the resource availability (e.g., inorganic nutrients and light) is limited to strict autotrophs (Wilken et al., 2014; Moeller et al., 2019). Although there is increasing recognition and understanding of the ecological significance of mixotrophic protists, it remains substantially understudied how phagotrophic algae take advantage of mixotrophic metabolisms in rapidly changing environments.

Phagotrophic algae are able to adjust their grazing activities according to the ambient inorganic nutrient concentrations. For instance, *Gyrodinium galatheanum* increases ingestion rate with decreasing inorganic phosphorus concentrations (Li et al., 2000; Smalley and Coats, 2002). Mixotrophic dinoflagellate *Heterocapsa triquetra* only ingests prey under nutrient-depleted conditions (Legrand et al., 1998). However, what the mixotrophic algae face in the changing ocean is not only the alteration in absolute inorganic nutrient concentrations but also the increasing imbalance in nutrient ratios due to anthropogenic activities (Li et al., 2000; Wickham and Wimmer, 2019).

The aquatic ecosystems have long been enriched with more N relative to P because of the sharp increase in the global use of N as well as the aggressive removal of P loads, which changes the nutrient stoichiometry and leads to the nutrient imbalance in aquatic ecosystems, especially in coastal waters (Huang et al., 2003; Glibert et al., 2013, 2014). Based on the framework of ecological stoichiometry, photoautotrophs have high plasticity in elemental composition and are apt to alter their elemental contents when the environment changes, whereas heterotrophic protists are prone to maintain the stoichiometry homeostatically (Sterner and Elser, 2002; Moreno and Martiny, 2018). Similar to heterotrophic protists, the phagocytosis of mixotrophs may enable them to maintain their cellular stoichiometry (Moorthi et al., 2017). Some studies have found that phagotrophic mixotrophs can regulate their phagotrophy in nutrient-imbalanced environments (Raven, 1997; Stoecker, 1998; Li et al., 2000; Smalley and Coats, 2002). For instance, the ingestion of *Gyrodinium galatheanum* increased as the ambient N:P ratios deviated from the Redfield ratio (Li et al., 2000).

The changing environmental N:P ratios also result in the variation of prey quality, affecting the ingestion activities of herbivorous consumers. To keep their elemental contents constant, consumers may adjust their feeding behaviour either through ingesting more favourable food to extract the limiting element more efficiently (i.e., selective feeding) or through an increase of food uptake and a reduction of handling time to extract the limiting nutrient only from the readily available parts (i.e., compensatory feeding; Knisely and Geller, 1986; Cruz-Rivera and Hay, 2000; Raubenheimer and Jones, 2006; Montagnes et al., 2008; Meunier et al., 2012). Such pre-gut selection mechanisms are usually assessed by the differences in food uptake. In addition, the consumers can use post-ingestion mechanisms (e.g., excretion of excess elements) to balance the nutrient-imbalanced food (Frost et al., 2005). The selective feeding behaviours have been widely observed in heterotrophic protists, such as *Oxyrrhis marina,* which can select P-rich prey from the mixture of prey with different N:P ratios (Montagnes et al., 2008; Meunier et al., 2012). Regarding mixotrophs, recent studies had found that mixotrophic flagellates in oligotrophic lakes showed prey preference when more than one kind of prey was available (Ballen-Segura et al., 2017; Gerea et al., 2019). Such prey preference was assumed to arise from different C:N:P ratios of prey (Ballen-Segura et al., 2017). However, more direct evidence is still needed for understanding how mixotrophs respond to prey with various nutritional qualities. Therefore, in the current study, we aim to investigate the selective feeding behaviours of mixotrophic protists and the potential influences of the ambient N:P ratio on their ingestion activities.

*Lepidodinium* sp. is a mixotrophic dinoflagellate isolated from subtropical coastal waters. It has the innate ability to photosynthesize but can ingest prey even under nutrient replete conditions. Their ingestion behaviour can be regulated according to the changes in inorganic nutrient concentrations (Liu et al., 2021). Our previous study also found that *Lepidodinium* sp. enhanced their ingestion rate and shifted towards more heterotrophy under warming conditions (Liu et al., 2021). If warming drives mixotrophs to behave more like heterotrophic protists, the effect of both inorganic nutrient concentrations and prey quality on the ingestion activities of mixotrophs could change accordingly. Thus, in this study, we used *Lepidodinium* sp. to investigate the selective feeding behaviour of phagotrophic algae under both nutrient-imbalanced and warming conditions. We conducted factorial experiments with three factors (i.e., inorganic N:P ratios, prey quality and temperature) to investigate (1) whether *Lepidodinium* sp. can undergo both compensatory feeding and selective feeding when provided prey of different nutritional quality; (2) how the inorganic N:P ratio affects the ingestion rate and prey selectivity of *Lepidodinium* sp.; and (3) whether rising temperature increases the ingestion rate of *Lepidodinium* sp. and enhances their prey selectivity.

## Materials and Methods

### Algae Cultures

*Lepidodinium* sp. used in this study was isolated from the Port shelter region of the Hong Kong Eastern area. Species identification was conducted by microscopic observation and 18 s rDNA sequencing. Based on BLAST search, our species showed 99% similarity to *Lepidodinium* sp. (MH 360; Ng et al., 2017). *Lepidodinium* sp. was grown under a 12:12 light cycle at 100 μmol photons m^−2^ s^−1^ in F/20 autoclaved filtered seawater medium. We added the *Rhodomonas salina,* which was grown in F/2 medium as prey, and checked the cultures every day to ensure sufficient prey for the *Lepidodinium* sp.

### Culture Pre-condition

The *Lepidodinium* sp. was grown and acclimated under different inorganic nutrient conditions, including Nitrogen-rich (N_high_, N:P ratio = 64), normal Nitrogen (N_Redfield_, N:P ratio = 16) and Nitrogen-limited (N_low_, N:P ratio = 4). The detailed nutrient concentrations are presented in Table 1. The *Rhodomonas salina* grown in F/2 medium was added to all cultures as prey for the *Lepidodinium* sp. All the above cultures with the three different inorganic nutrient conditions were acclimated at 25, 28 and 31°C for at least 2 weeks. We used semi-continuous cultures (i.e., transfer every 4 days) during the acclimation to keep the *Lepidodinium* sp. growing in an exponential growth phase.

**Table 1:** The nitrate (N) and phosphorus (P) concentrations (μmol L^−1^) of the culture medium used for *Lepidodinium* sp. culture mediums.

	Inorganic N	Inorganic P	N:P ratio
**N**_high_	175	2.7	64
**N**_Redfield_	175	10.9	16
**N**_low_	43.8	10.9	4

### Selective Feeding Experiments

We have conducted two rounds of selective feeding experiments. One round was carried out under three inorganic nutrient conditions at 25°C to investigate the ingestion behaviour of the mixotrophic *Lepidodinium* sp. The other round was conducted under three inorganic nutrient conditions at three temperatures (25, 28 and 31°C) to investigate the combined effect of temperature and inorganic nutrients on the ingestion behaviour of *Lepidodinium* sp.

The prey with different nutrition values was prepared by cultivating the *Rhodomonas salina* in the nitrogen-repleted and nitrogen-depleted mediums. The F/2 medium and F/2 medium without adding NO_3_^−^ were used for the high-N prey and low-N prey, respectively. All cultures were maintained under the same light condition (100 μmol photons m^−2^ s^−1^) at 22°C. The *Rhodomonas salina* cells during the stationary phase were used in the selective feeding experiments to ensure a significant difference between high-N and low-N preys. It has been found that the colour of *Rhodomonas salina* cultures grown in nitrogen-limited mediums turned to be green or yellow, which is different from the cultures in nitrogen-replete mediums (red). This difference is caused by a lack of phycoerythrin in nitrogen-limited *Rhodomonas salina* cells (John and Davidson, 2001). It can be distinguished by flow cytometry with different fluorescence signals (Supplementary Figure S1). The cellular contents and C:N:P ratios were also significantly different when grown in these two mediums, while the cell size was similar (Table 2; Supplementary Figure S1), which renders the *Rhodomonas salina* as ideal prey for selective feeding experiments (Meunier et al., 2016).

**Table 2:** The N and P contents (mean of four replicates ± SD) of *Rhodomonas salinia* cultured in F/2 medium and F/2 medium without N addition, respectively.

Culture medium	N (pg cell^−1^)	P (pg cell^−1^)	N:P ratio
F/2	13.19 ± 0.71	0.71 ± 0.04	41
F/2 without N	6.59 ± 0.26	0.96 ± 0.03	15

All data were from (Meunier et al., 2016).

The selective feeding experiments were designed as feeding the *Lepidodinium* sp. grown in three nutrient conditions with mixed preys (high-N: low-N = 1:1). Before the selective feeding experiments, the *Lepidodinium* sp. cultures were starved for 2 days to ensure all *Rhodomonas salina* in the pre-conditioned cultures had been consumed and the food vacuole of *Lepidodinium* sp. was emptied, which minimised the influence of undigested food on the grazing selectivity of grazers. The *Lepidodinium* sp. cultures were starved for 1 day in the first-round experiments conducted at 25°C.

Upon the experiments, the *Lepidodinium* sp. cultures were filtered through 10 μm polycarbonate membrane filters (GVS Corporation) and resuspended into nutrient-free sterile artificial seawater to eliminate the remaining nutrient and prey. The high-N and low-N *Rhodomonas salina* used in the selective feeding experiments were centrifuged with a speed of 800 rpm for 5 min at 22°C and then washed and resuspended in nutrient-free autoclaved artificial seawater. The *Lepidodinium* sp. and *Rhodomonas salina* were observed under a microscope to guarantee that they were in good status after the treatments. The *Lepidodinium* sp. with a final concentration of 500 cells ml^−1^ (~1,000 cells ml^−1^ in first-round experiments) were transferred to 10 ml mediums with sufficient prey (100,000 ~ 160,000 cell ml^−1^). The nutrient concentration of the experimental mediums was the same as the pre-conditions. For instance, the N:P ratio of the experimental medium was set to be 16 when the ratio of pre-condition was 16. The prey was a mixture of high-N and low-N *Rhodomonas salina* in a ratio of 1:1. The control groups were set up with the same nutrient and prey concentrations but without grazers. All experimental and control groups were set up in triplicates. To prevent dramatic changes in nutrient concentrations and the depletion of prey, the grazing experiments lasted for 6 h (Båmstedt et al., 2000; Wilken et al., 2013), and the prey with different N contents (i.e., high-N prey and low-N prey) can be well distinguished after 6 h incubation (Supplementary Figure S1). In the first-round experiment conducted at 25°C, samples for measuring prey and predator concentrations were collected at four time points (0, 2, 4, 6 h), while in the second-round experiments conducted at three temperatures, the samples were collected only at 0 and 6 h of each experiment. Subsamples (2 ml) for counting the cell abundance of *Lepidodinium* sp. were collected, fixed by Lugol’s solution (final concentration 2%) and observed under a microscope. Other subsamples (1.8 ml) for prey concentration were collected, fixed by 50 μl 20% paraformaldehyde solution (0.5% final concentration) and analysed using a Becton-Dickinson FACSCalibur flow cytometer. The samples were run for 5 min at a high flow rate (57 ~ 60 μl/min) to enable sufficient events for calculations and minimise the measurement errors.

To compare the ingestion responses of mixotrophic species and heterotrophic species, we conducted another extra selective feeding experiment at 25°C using a heterotrophic species, *O. marina.* The experimental treatments of medium conditions and prey quality were the same as the abovementioned for *Lepidodinium* sp.

### Estimate of Ingestion Rate and Prey Selectivity Index

Ingestion rates of *Lepidodinium* sp. and *O. marina* (*I*, prey predator h^−1^) in grazing experiments were calculated referring to the formula (Båmstedt et al., 2000):


(1) $$I=\frac{C_{0}-C_{t}+\left[C\right]\times \left(e^{k\times t}-1\right)}{n\times t}$$

(2) $$k=\frac{1}{t}\ln\left(\frac{R_{t}}{R_{0}}\right)$$

where *C_0_* and *C_t_* are the prey concentrations at the beginning and end of the experiment (i.e., 0 and 6 h), respectively; [C] is the mean prey concentration; *t* is incubation time; *n* is the cell concentration of *Lepidodinium* sp. we set in the experiment as the *Lepidodinium* sp. concentration did not vary a lot over 6 h incubation (Supplementary Figure S2); and *k* is the instantaneous growth coefficient of the prey calculated by Eq. (2). In Eq. (2), *R_t_* and *R_0_* are the cell abundances of *Rhodomonas salina* in the control bottles without grazers at the end and beginning of the experiments, respectively. The ingestion rates for high-N and low-N preys were calculated using the corresponding prey concentrations at the beginning and end of experiments, respectively. This equation assumes a linear food reduction as a function of incubation time, which is confirmed by the trajectory of prey in the first-round experiments (Supplementary Figure S2). We then estimated the ingestion using Eq. (1) based on the reasonable prey reduction over 6 h incubation (Supplementary Figures S2, S3).

To evaluate the selectivity of *Lepidodinium* sp. towards the *Rhodomonas salina* with two different nutrition qualities, we calculated Manly’s α preference index, which compares the proportion of one prey in the diet with its proportion in the environment and is also known as Chesson’s index (Manly, 1974; Chesson, 1978, 1983):


(3) $$\alpha =\frac{\frac{r_{i}}{n_{i}}}{\sum_{i=1}^{m}\frac{r_{i}}{n_{i}}}$$

where *r_i_* was the proportion of prey *i* in the diet, *n_i_* was the proportion of prey *i* in the environment, which was calculated by the abundance of prey *i* divided by the total sum of available prey abundance. The selectivity index α varies between 0 and 1, and *α_i_* = 0.5 indicated nonselective feeding towards the prey *i*. The Chesson’s index has been widely used to evaluate the prey selectivity of predators in aquatic ecosystems, such as insects (e.g., Klecka and Boukal, 2012), zooplankton (e.g., Meunier et al., 2016) and heterotrophic dinoflagellates (e.g., Hansen and Calado, 1999; Meunier et al., 2012).

### Statistical Analysis

All data are expressed as the mean ± SD unless otherwise indicated. Differences in ingestion rate and prey selectivity among the treatments were tested using one-way ANOVA after grouping data by either temperature or nutrient condition. The Tukey’s Honest Significant Difference test (i.e., Tukey HSD test) was conducted following one-way ANOVA to examine the difference between specific groups by comparing all possible pairs of means. The effect of temperature and nutrient condition on ingestion rate and selectivity index was examined by two-way ANOVA. The student’s *t*-test was used to investigate whether the Chesson selectivity index α is significantly different from 0.5. All analyses were considered significant at *p* < 0.05 and conducted using GraphPad Prism (Version 8.3.0.) and R 3.4.3 (R Core Team, 2017).

## Results

### Compensatory Feeding and Selective Feeding Behaviour of *Lepidodinium* sp.

**Figure 1:**
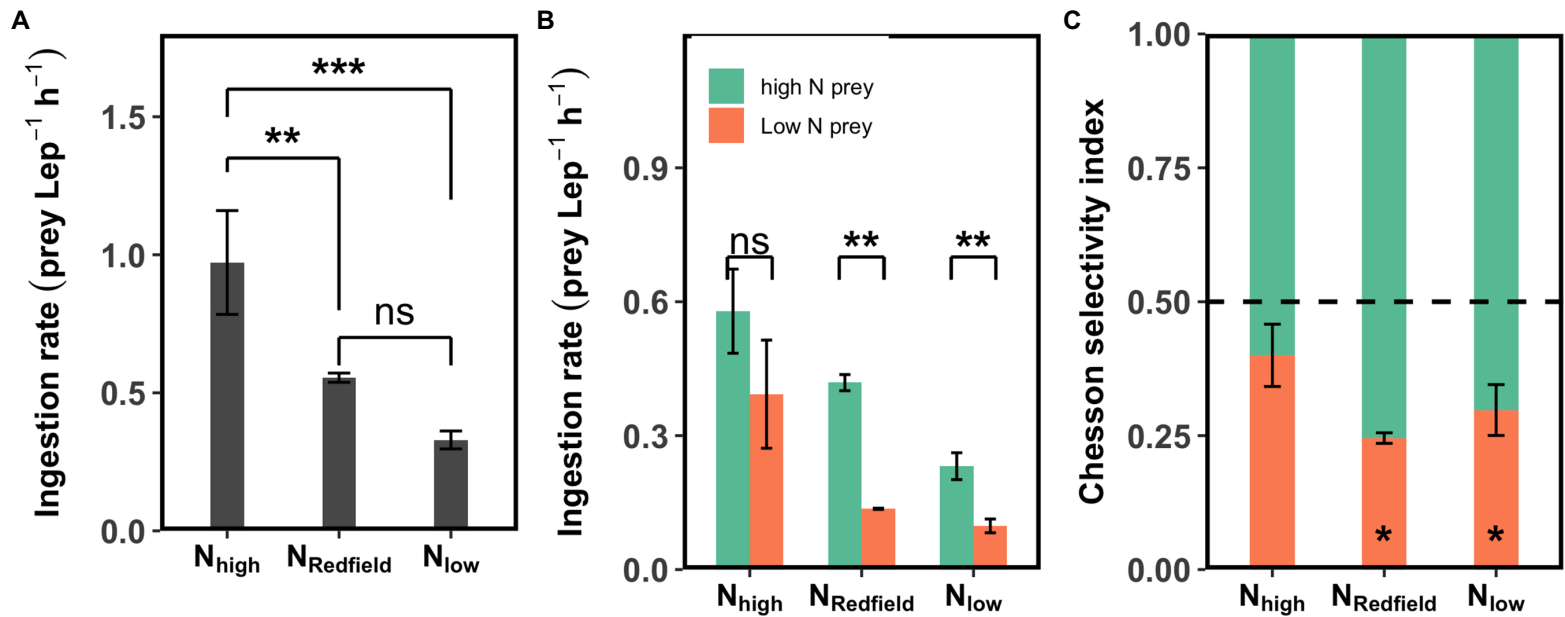
The results of first-round selective feeding experiments conducted at 25°C. The total ingestion rate **(A)**, the ingestion rate on high-N and low-N prey **(B)**, and the Chesson selectivity index **(C)** of *Lepidodinium* sp. under N_high_ (N:P ratio = 64), N_Redfield_ (N:P ratio = 16), and N_low_ (N:P ratio = 4) conditions at 25°C. The asterisk in **(A)** and **(B)** indicates the significant difference between specific groups, e.g., the high-N prey and low-N prey (Tukey HSD Test following a one-way ANOVA); the asterisk in **(C)** shows the difference of Chesson selectivity index from 0.5 (Student’s *t*-test; ^*^p < 0.05; ^**^p < 0.01; ^***^p < 0.001; and ns: *p* > 0.05).

The total ingestion rate of *Lepidodinium* sp. on *Rhodomonas salina* (high-N + low-N) under N_Redfield_ (N:P = 16) and N_low_ (N:P = 4) conditions was significantly lower than the N_high_ (N:P = 64) conditions at 25°C in the first-round experiments (Tukey HSD test, *p* < 0.01; Figure 1A), which indicates that the *Lepidodinium* sp. may conduct compensatory feeding under N_high_ condition at 25°C. In this experiment, the ingestion rate of *Lepidodinium* sp. on high-N prey was significantly higher than on low-N prey under N_Redfield_ and N_low_ conditions where the nitrogen could be limited for *Lepidodinium* sp. (Tukey HSD test, *p* < 0.01; Figure 1B). Under these two situations (N_Redfield_ and N_low_), *Lepidodinium* sp. exhibited selective feeding behaviour towards high-N prey, with the Chesson selectivity index α of 0.75 ± 0.01 and 0.70 ± 0.05, respectively, which were significantly different from 0.5 (Student’s *t*-test, *p* < 0.05; Figure 1C). By contrast, the ingestion rate on high-N prey and low-N prey was not significantly different when the inorganic N was sufficient with the N:P ratio of 64 (Tukey HSD test, *p* > 0.05; Figure 1B), although the Chesson selectivity index α is a bit higher than 0.5 (0.60 ± 0.06; *p* > 0.05; Figure 1C).

**Figure 2:**
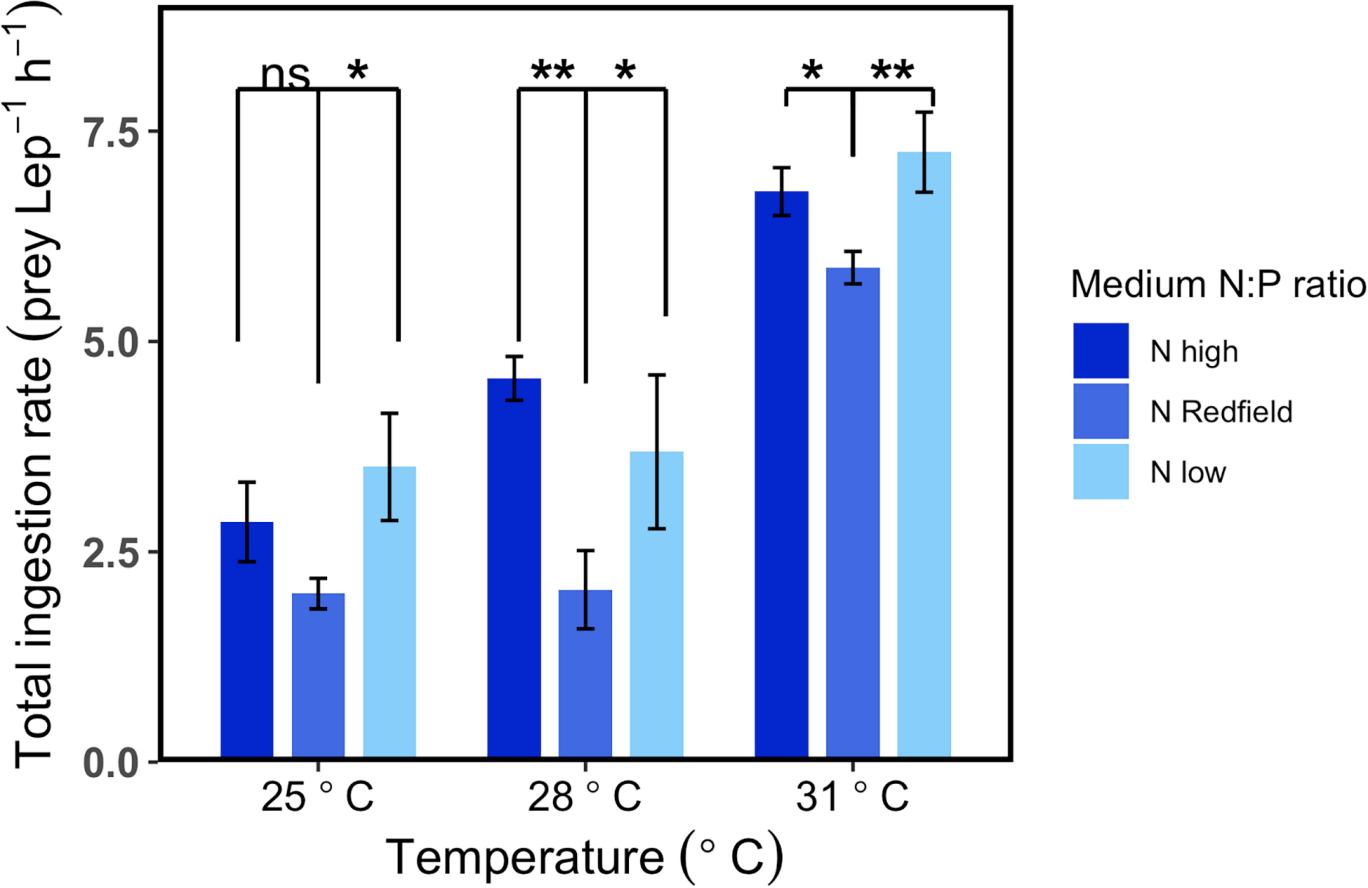
Total ingestion rate of *Lepidodinium* sp. under three different nutrient treatments (N_high_, N_Redfield_ and N_low_) at three different temperatures (25, 28 and 31°C) in the second-round experiments. The asterisk indicates significant difference from N_Redfield_ conditions at each temperature (Tukey HSD Test following a one-way ANOVA; ^*^p < 0.05; ^**^p < 0.01; ^***^p < 0.001; and ns: *p* > 0.05).

**Figure 3:**
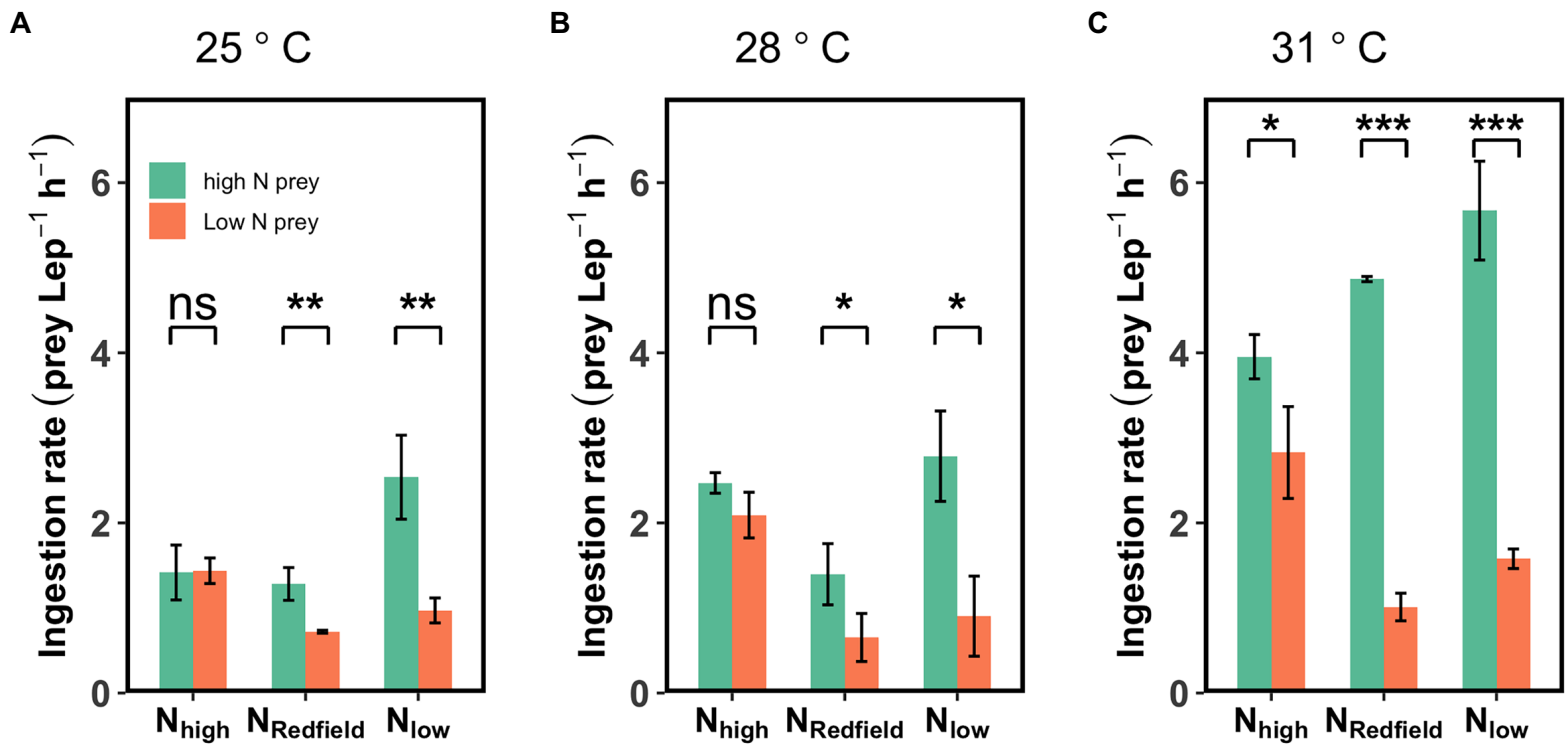
Ingestion rate of *Lepidodinium* sp. on high-N prey and low-N prey under three different nutrient treatments (N_high_, N_Redfield_ and N_low_) at 25°C **(A)**, 28°C **(B)** and 31°C **(C)**. The asterisk indicates the significant difference between high-N prey and low-N prey (Tukey HSD Test following a one-way ANOVA; ^*^p < 0.05; ^**^p < 0.01; ^***^p < 0.001; and ns: *p* > 0.05).

The compensatory feeding and selective feeding behaviours were also observed at three temperatures in the second-round experiments. The patterns showed at 25°C in the two rounds of experiments were similar (Figures 1, 2, 3A), although the ingestion rate was lower in the first-round experiments because the initial concentration of *Lepidodinium* sp. was higher, and they were starved for only 1 day before experiments. At 28 and 31°C, the total ingestion rates under N_high_ and N_low_ conditions were significantly higher than N_Redfield_ conditions (Tukey HSD test, *p* < 0.05; Figure 2). The significant higher total ingestion rate under N_high_ and N_low_ conditions were also observed at 19, 22 and 25°C in our preliminary experiments (Supplementary Figure S4), suggesting that *Lepidodinium* sp. ingest more prey when the inorganic nutrient ratio was imbalanced.

At 28°C, the difference between the ingestion rate on high-N prey and low-N prey was significant under N_Redfield_ and N_low_ conditions (Tukey HSD test; *p* < 0.05) but not the N_high_ conditions (*p* > 0.05; Figure 3B). While at 31°C, the selective feeding behaviour of *Lepidodinium* sp. not only occurred under N_Redfield_ and N_low_ but also under N_high_ conditions, as the ingestion rate on high-N prey was significantly higher than on low-N prey (Tukey HSD test; *p* < 0.05; Figure 3C).

In comparison with the mixotrophic *Lepidodinium* sp., although the heterotrophic dinoflagellate *O. marina* had a higher ingestion rate, they did not exhibit selective feeding on the *Rhodomonas salina* under three nutrient conditions. The Chesson selectivity index α was all about 0.5 (0.48 ± 0.05, 0.46 ± 0.07 and 0.45 ± 0.02 for N_high_, N_Redfield_ and N_low_, respectively; Supplementary Figure S5).

### Effect of Temperature on the Total Ingestion Rate and Selective Feeding of *Lepidodinium* sp.

The total ingestion rate increased with increasing temperature under the three inorganic nutrient conditions (Figure 2; Supplementary Figure S4). The total ingestion rate of *Lepidodinium* sp. was affected by both temperature (two-way ANOVA, *p* < 0.001) and environmental inorganic nutrient conditions (two-way ANOVA, *p* < 0.01, Table 3).

**Table 3:** Summary of the two-way ANOVA analysis on the effects of medium N:P ratios and temperature on the ingestion rate of different prey (high N and low N) and prey selectivity of *Lepidodinium* sp.

Parameter	Factor	df1	df2	Single effect		Factor interaction	
				*F*	*P*	*F*	*P*
Ingestion rate on high-N prey	NP ratio	2	21	6.64	0.0058	0.79	0.4678
	Temperature	1	21	77.94	<0.0001		
Ingestion rate on low-N prey	NP ratio	2	21	43.23	<0.0001	5.17	0.0149
	Temperature	1	21	27.58	<0.0001		
Prey selectivity (α)	NP ratio	2	21	43.42	<0.0001	2.91	0.0767
	Temperature	1	21	20.58	<0.0001		
Total ingestion rate	NP ratio	2	21	7.84	0.0029	0.02	0.9834
	Temperature	1	21	81.09	<0.0001		

**Figure 4:**
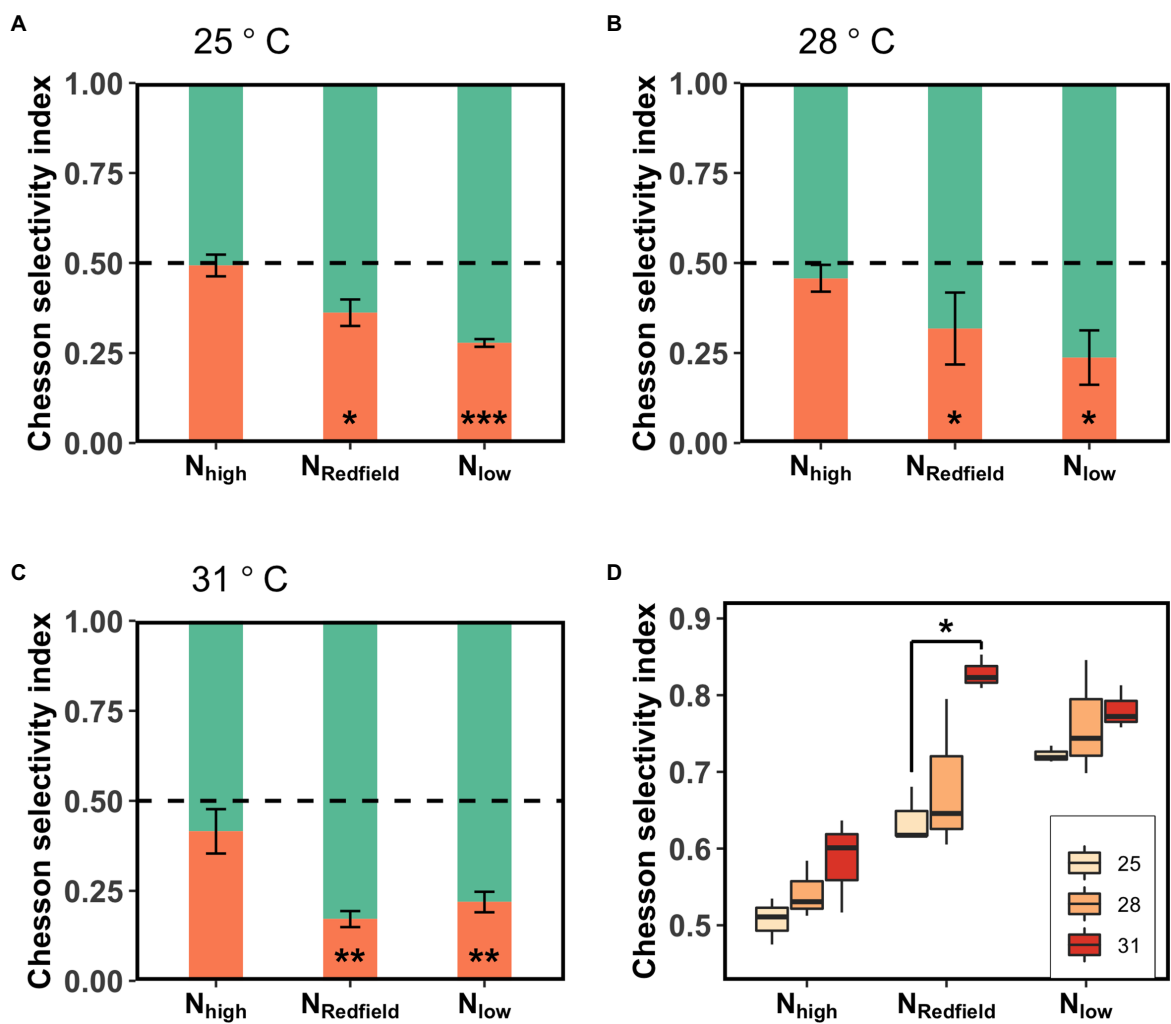
The Chesson selectivity index of *Lepidodinium* sp. under three different nutrient treatments (N_high_, N_Redfield_ and N_low_) at 25°C **(A)**, 28°C **(B)** and 31°C **(C)**. The green and orange bars represent the Chesson selectivity index towards high-N prey and low-N prey, respectively. The asterisk shows the difference of Chesson selectivity index from 0.5 (Student’s *t*-test; ^*^p < 0.05; ^**^p < 0.01; ^***^p < 0.001; and ns: *p* > 0.05). The data grouped by nutrient treatments was showed in **(D)**. The asterisk indicates the significant difference between two temperatures (Tukey HSD Test following a one-way ANOVA).

Increasing temperature not only increased the total ingestion rate of the *Lepidodinium* sp. but also exacerbated their feeding selectivity (Figures 3, 4). Under N_Redfield_ and N_low_ conditions, the ingestion rate of *Lepidodinium* sp. on the low-N prey remained unchanged at the three temperatures (one-way ANOVA, *p* > 0.05, Figure 3; Supplementary Figure S6). Nevertheless, the ingestion rate on high-N prey significantly increased when temperature increased from 28°C to 31°C under N_Redfiled_ and N_low_ conditions (Tukey HSD test; *p* < 0.05; Figure 3; Supplementary Figure S6). Therefore, the Chesson’s selectivity index α increased significantly from 0.64 ± 0.1 to 0.83 ± 0.02 under N_Redfiled_ condition (Tukey HSD test; *p* < 0.0001; Figure 4). While rising temperature did not significantly affect prey selectivity of *Lepidodinium* sp. under N_low_ condition because the selectivity index kept high (one-way ANOVA, *p* > 0.05; Figure 4). The Chesson selectivity index α was 0.76 ± 0.08 and 0.78 ± 0.03 under N_low_ conditions at 28°C and 31°C, respectively (Figure 4). Under the N_high_ condition, *Lepidodinium* sp. increased their ingestion on both high-N and low-N prey when the temperature increased (Figure 3; Supplementary Figure S6). However, the increase in the ingestion rate on high-N prey was more significant, resulting in selective feeding and a slight increase in the Chesson’s selectivity index α at 31°C (0.58 ± 0.06; Figure 4).

The prey selectivity of *Lepidodinium* sp. was significantly regulated by both temperature (two-way ANOVA, *p* = 0.012 < 0.05) and pre-conditions with different medium N:P ratios (two-way ANOVA, *p* < 0.0001; Table 3).

## Discussion

Although mixotrophs have long been recognised as widespread and critical components in planktonic communities and the aquatic food web (Mitra et al., 2016; Stoecker et al., 2017), how they respond to changing environments remains substantially understudied. In the current study, we investigated the ingestion activities of a mixotrophic dinoflagellate under various conditions with strong implications relevant to the response of similar mixotrophic protists to warming and the growing nutrient imbalance in aquatic ecosystems.

### Compensatory Feeding and Selective Feeding of Mixotrophic Dinoflagellate Under Nutrient-Imbalanced Conditions

*Lepidodinium* sp. is a facultative phagotrophic dinoflagellate, of which photosynthesis is obligate and phagotrophy is facultative (Liu et al., 2021). Different from many mixotrophic algae that consume prey when inorganic nutrients are limited, *Lepidodinium* sp. can ingest prey even when nutrients are sufficient for their photosynthesis (Liu et al., 2021). Our study also observed the ingestion behaviours under N_Redfiled_ conditions in which the nutrient concentrations (~20% f/2 medium) did not limit the growth of *Lepidodinium* sp. (Liu et al., 2021). This result suggests that the ingestion is advantageous to *Lepidodinium* sp. for more reasons than the supply of N or P. For instance, the ingestion could be the means for maintaining the internal stoichiometric balance (Stoecker et al., 2017). Moreover, *Lepidodinium* sp. increased their ingestion rate significantly when the inorganic N:P ratio in ambient water deviated from the Redfield ratio (i.e., N_high_ and N_low_ conditions; Figures 1A, 2; Supplementary Figure S4). As the inorganic N:P ratios were achieved by manipulating either N or P concentrations, the increase in the ingestion rate could arise from the potential N or P limitations. It is likely that *Lepidodinium* sp. ingested more prey to acquire more P under N_high_ conditions and more N under N_low_ conditions. The acquisition of P or N through enhanced ingestion then supports photosynthesis and growth. Thus, mixotrophy not only benefits the acquisition of carbon but also provides an important channel to replenish the nutrients (Glibert and Burkholder, 2011).

However, another explanation for the increasing ingestion rate under nutrient-imbalanced conditions is the lopsided N:P ratio rather than the limiting factors. In fact, the nutrient concentrations we used (2.7 μmol L^−1^ P for N_high_ and 43.8 μmol L^−1^ N for N_low_) were actually sufficient for *Lepidodinium* sp. to grow autotrophically (Liu et al., 2021). As such, the upregulation in the ingestion rate of *Lepidodinium* sp. may be for the sake of maintaining the cellular elemental balance, which has also been observed in other mixotrophic dinoflagellates such as *Gyrodinium galatheanum* and *Prymnesium parvum*. They also increased their feeding rate when grown under imbalanced N:P ratio conditions (Li et al., 2000; Lundgren et al., 2016). The feeding strategy is similar to the ‘compensatory feeding’ of heterotrophic consumers, which increase food uptake to extract the limiting nutrients only from the readily available parts (Raubenheimer and Jones, 2006; Montagnes et al., 2008; Meunier et al., 2012).

In addition to the ‘compensatory feeding’ behaviour, we also found that *Lepidodinium* sp. exhibits significant selective feeding behaviour towards N-rich prey under N_Redfiled_ and N_low_ conditions (Figures 1, 3, 4). Although the inorganic nutrient concentrations of N_Redfiled_ condition were sufficient for the growth of *Lepidodinium* sp. (Liu et al., 2021), the N:P ratio may not be optimal, which renders *Lepidodinium* sp. to graze more N-rich prey to achieve the cellular optimal N:P ratios. Under the N_low_ condition, the relatively low N may not satisfy the demand of *Lepidodinium* sp.; therefore, they grazed more N-rich prey to extract the limiting element more efficiently. It is also likely that the relatively low N disrupts the balance of the cellular stoichiometry of *Lepidodinium* sp., driving them to ingest more N to achieve the balance. By contrast, we did not observe the selective feeding behaviour of *Lepidodinium* sp. under N_high_ condition at 25 and 28°C because the inorganic N was in excess, and they do not need to ingest more N to supplement photosynthesis or for stoichiometric balance. Nevertheless, it could be possible that there was less P or P limitation under N_high_ condition, so *Lepidodinium* sp. fed more *Rhodomonas salina* to extract P contents (Figures 2, 3). Whereas the grazing was indiscriminate because the P contents of the two kinds of prey were similar (Table 2). Therefore, the selective feeding behaviour of mixotrophic protists is regulated by both inorganic nutrient conditions and the prey quality.

For comparison, we also examined the feeding behaviours of a heterotrophic protist (i.e., *O. marina*) under the same conditions. As the stoichiometric balance of heterotrophic protists mainly depends on ingestion activities, they may be more susceptible to prey quality and environmental changes. Nevertheless, we did not observe any selective feeding of *O. marina* under three N:P ratio conditions (Supplementary Figure S5). Also, prey with different N contents did not trigger their selective feeding behaviour. The result is consistent with a previous study, which found selective feeding of *O. marina* on P-rich prey but not on N-rich prey (Meunier et al., 2012). The results of the comparison suggest that mixotrophic protists might be more apt to selectively feed on different prey due to the influence of inorganic nutrients, which may help them adapt to the environmental changes. The ability of selective feeding and compensatory feeding may endow them with a competitive edge in nutrient-limited and -imbalanced environments. In such environments, the growth rate of mixotrophic protists could be higher than the comparable autotrophic and heterotrophic ones (Lundgren et al., 2016; Lin et al., 2018). Thus, the mixotrophs could become increasingly important in aquatic environments, especially in coastal waters with increasing nutrient imbalance (Mitra et al., 2014).

The feeding strategy (i.e., selective feeding or compensatory feeding) is commonly used by metazoan in aquatic ecosystems such as copepods (Boersma et al., 2016; Meunier et al., 2016) and *Daphnia* sp. (DeMott, 1998), which is conducive to compensate the elemental or biochemical deficiencies. The feeding behaviour of consumers strongly influences the environmental nutrient composition and prey community, such as the phytoplankton (Elser and Urabe, 1999; Vanni, 2002). Our study substantiated that phagotrophic algae also have the ability to use feeding strategy to compensate for the elemental deficiencies to maintain their internal balance, which gives feedback to the environments at the same time. By selectively feeding on the scarce elements, for instance, feeding more N-rich prey in low N:P ratio environments, *Lepidodinium* sp. may remove the limiting element from the phytoplankton community, which may indirectly reshape the community. Although the same prey with different nutritional contents designed in our experiments rarely occurs in nature, our results suggest that mixotrophic protists may selectively feed on prey with various C:N:P ratios under different conditions, potentially influencing the plankton community and food web structure (Ballen-Segura et al., 2017; Gerea et al., 2019).

### Warming Exacerbates Selective Feeding Towards High-N Prey

The increase of nutrient imbalance in aquatic ecosystems is usually accompanied by temperature rise under the context of climate changes. In the current study, we examined the effect of nutrient imbalance on mixotrophs at different temperatures and found that warming exacerbates the selective feeding activities of *Lepidodinium* sp. (Figures 3, 4). This could be a result of the increasing contribution of ingestion activity to the growth of *Lepidodinium* sp. (Liu et al., 2021). Several empirical evidence has proved that warming shifts mixotrophs towards more heterotrophic (Wilken et al., 2013; Liu et al., 2021). According to the metabolic theory of ecology (i.e., MTE; Allen et al., 2005; Chen et al., 2012; Liu et al., 2019), heterotrophic ingestion is more sensitive to temperature changes than autotrophic photosynthesis. As such, phagotrophy would become more important for mixotrophs as temperature increases. In the current study, we observed that *Lepidodinium* sp. has significantly increased its ingestion rate to satisfy its growth demand (Figure 2; Supplementary Figure S4). Moreover, the increases in the ingestion rate with increasing temperature were all contributed by the ingestion on the high-N prey, while the ingestion on the low-N prey remained unchanged, leading to higher Chesson’s selectivity index under N_Redfiled_ and N_low_ conditions (Figure 4). As warming may change the internal N:P ratios and break the balance (Thrane et al., 2017), *Lepidodinium* sp. tended to graze more N-rich food to achieve stoichiometry balance as temperature increased (Figure 3). Thus, warming exacerbates *Lepidodinium* sp.’s selective feeding towards high-N prey.

Also, under the high N:P ratio condition, *Lepidodinium* sp. started to show selective feeding behaviour when temperature increased (Figures 3, 4). Although the demand for N for growth increased with increasing temperature, the concentration we set was relatively high (175 μmol/l, Table 1), which should be sufficient for *Lepidodinium* sp., even at high temperatures. It is possible that nitrogen uptake and photosynthesis of *Lepidodinium* sp. are constrained at high temperatures. In our previous study, we found that the autotrophic growth determined by photosynthesis was significantly lower than the mixotrophic growth determined by both ingestion and photosynthesis at high temperatures, which suggested that photosynthesis may be constrained by high temperature and phagocytosis contributed more to the growth of the mixotrophs (Liu et al., 2021). To maintain the stoichiometric balance that may be shifted by the increase of temperature, *Lepidodinium* sp. ingested more N-rich food to compensate for the deficiency of photosynthesis. That may explain the selective feeding towards high-N prey of mixotrophic protists even under inorganic N replete conditions as temperature increases, which points to an increase in heterotrophic activity of mixotrophs in the warmer ocean.

## Conclusion

In the past four decades, seawater N:P ratios in many coasts and estuaries increased dramatically due to increasing nitrogen fertiliser and domestic and industrial wastewater discharge (Glibert et al., 2013, 2014). This might cause an increase in the ingestion rate of mixotrophs with a similar ecological niche as *Lepidodinium* sp. in the future and subsequently shift their functional role from primary producers to consumers (Mitra et al., 2014; Leles et al., 2018). Moreover, selective feeding towards prey with high nutritional quality may influence the community composition of phytoplankton. A warmer ocean could exacerbate such trends that cause the mixotrophs to become more heterotrophic and consequently change the structure of the planktonic food web and carbon and nutrient cycling in marine ecosystems. It should be pointed out that there are many types of mixotrophs, from highly autotrophic to highly heterotrophic (Stoecker, 1998; Mitra et al., 2016; Stoecker et al., 2017). More studies should focus on the response of different types of mixotrophic species to various environmental factors to elucidate and predict the ecological roles of the mixotrophs in marine ecosystems under projected climate warming.

## Data Availability Statement

The raw data supporting the conclusions of this article will be made available by the authors, without undue reservation.

## Author Contributions

HN, KL and HL conceived the study and designed the experiments. HN conducted the experiments. ZG helped repeated the experiments. KL and HN wrote the draft. KL and HL revised and improved the manuscript. All authors contributed to the article and approved the submitted version.

## Funding

This study was supported by the Research Grants Council of Hong Kong (16101318 and T21/602/16), the Key Special Project for Introduced Talents Team of Southern Marine Science and Engineering Guangdong Laboratory (Guangzhou) (GML2019ZD0409), and the Hong Kong Branch of the Southern Marine Science and Engineering Guangdong Laboratory (Guangzhou; SMSEGL20SC01).

## Conflict of Interest

The authors declare that the research was conducted in the absence of any commercial or financial relationships that could be construed as a potential conflict of interest.

## Publisher’s Note

All claims expressed in this article are solely those of the authors and do not necessarily represent those of their affiliated organizations, or those of the publisher, the editors and the reviewers. Any product that may be evaluated in this article, or claim that may be made by its manufacturer, is not guaranteed or endorsed by the publisher.

## References

[ref1] Allen A. P. Gillooly J. F. Brown J. H. (2005). Linking the global carbon cycle to individual metabolism. Funct. Ecol. 19, 202–213. doi: 10.1111/j.1365-2435.2005.00952.x

[ref2] Ballen-Segura M. Felip M. Catalan J. (2017). Some mixotrophic flagellate species selectively graze on archaea. Appl. Environ. Microbiol. 83, e02317–e02316. doi: 10.1128/AEM.02317-16 27815273 PMC5203632

[ref3] Båmstedt U. Gifford D. J. Irigoien X. Atkinson A. Roman M. (2000). “Feeding,” in ICES Zooplankton Methodology Manual. eds. Harris R. Wiebe P. Lenz J. Skjoldal H. R. Huntley M. (Unites States: Academic Press), 297–380.

[ref5] Boersma M. Mathew K. A. Niehoff B. Schoo K. L. Franco-Santos R. M. Meunier C. L. (2016). Temperature driven changes in the diet preference of omnivorous copepods: no more meat when it’s hot? Ecol. Lett. 19, 45–53. doi: 10.1111/ele.12541, PMID: 26567776

[ref7] Chen B. Landry M. R. Huang B. Liu H. (2012). Does warming enhance the effect of microzooplankton grazing on marine phytoplankton in the ocean? Limnol. Oceanogr. 57, 519–526. doi: 10.4319/lo.2012.57.2.0519

[ref8] Chesson J. (1978). Measuring preference in selective predation. Ecology 59, 211–215. doi: 10.2307/1936364

[ref9] Chesson J. (1983). The estimation and analysis of preference and its relationship to foraging models. Ecology 64, 1297–1304. doi: 10.2307/1937838

[ref10] Cruz-Rivera E. Hay M. E. (2000). Can quantity replace quality? Food choice, compensatory feeding, and fitness of marine mesograzers. Ecology 81, 201–219. doi: 10.1890/0012-9658(2000)081[0201:CQRQFC]2.0.CO;2

[ref11] DeMott W. R. (1998). Utilization of a cyanobacterium and a phosphorus deficient green alga as complementary resources by daphnids. Ecology 79, 2463–2481. doi: 10.1890/0012-9658(1998)079[2463:UOACAA]2.0.CO;2

[ref12] Elser J. J. Urabe J. (1999). The stoichiometry of consumer driven nutrient recycling: theory, observations, and consequences. Ecology 80, 735–751. doi: 10.1890/0012-9658(1999)080[0735:TSOCDN]2.0.CO;2

[ref13] Flynn K. J. Mitra A. (2009). Building the “perfect beast”: modelling mixotrophic plankton. J. Plankton Res. 31, 965–992. doi: 10.1093/plankt/fbp044

[ref14] Frost P. C. Evans-White M. A. Finkel Z. V. Jensen T. C. Matzek V. (2005). Are you what you eat? Physiological constraints on organismal stoichiometry in an elementally imbalanced world. Oikos 109, 18–28. doi: 10.1111/j.0030-1299.2005.14049.x

[ref15] Gerea M. Queimaliños C. Unrein F. (2019). Grazing impact and prey selectivity of picoplanktonic cells by mixotrophic flagellates in oligotrophic lakes. Hydrobiologia 831, 5–21. doi: 10.1007/s10750-018-3610-3

[ref16] Glibert P. M. Burkholder J. M. (2011). Harmful algal blooms and eutrophication: “strategies” for nutrient uptake and growth outside the Redfield comfort zone. Chin. J. Oceanogr. Limnol. 29, 724–738. doi: 10.1007/s00343-011-0502-z

[ref17] Glibert P. M. Kana T. M. Brown K. (2013). From limitation to excess: consequences of substrate excess and stoichiometry for phytoplankton physiology, trophodynamics and biogeochemistry, and implications for modeling. J. Mar. Syst. 125, 14–28. doi: 10.1016/j.jmarsys.2012.10.004

[ref18] Glibert P. M. Maranger R. Sobota D. J. Bouwman L. (2014). The Haber Bosch –harmful algal bloom (HB-HAB) link. Environ. Res. Lett. 9:105001. doi: 10.1088/1748-9326/9/10/105001

[ref19] Hansen P. J. Calado A. J. (1999). Phagotrophic mechanisms and prey selection in free-living dinoflagellates^1^. J. Eukaryot. Microbiol. 46, 382–389. doi: 10.1111/j.1550-7408.1999.tb04617.x

[ref20] Huang X. Huang L. Yue W. (2003). The characteristics of nutrients and eutrophication in the Pearl River estuary, South China. Mar. Pollut. Bull. 47, 30–36. doi: 10.1016/S0025-326X(02)00474-5, PMID: 12787594

[ref21] John E. Davidson K. (2001). Prey selectivity and the influence of prey carbon: nitrogen ratio on microflagellate grazing. J. Exp. Mar. Biol. Ecol. 260, 93–111. doi: 10.1016/S0022-0981(01)00244-1, PMID: 11358573

[ref22] Klecka J. Boukal D. S. (2012). Who eats whom in a pool? A comparative study of prey selectivity by predatory aquatic insects. PLoS One 7:e37741. doi: 10.1371/journal.pone.0037741, PMID: 22679487 PMC3367957

[ref23] Knisely K. Geller W. (1986). Selective feeding of four zooplankton species on natural lake phytoplankton. Oecologia 69, 86–94. doi: 10.1007/BF00399042, PMID: 28311689

[ref24] Legrand C. Graneli E. Carlsson P. (1998). Induced phagotrophy in the photosynthetic dinoflagellate Heterocapsa triquetra. Aquat. Microb. Ecol. 15, 65–75. doi: 10.3354/ame015065

[ref25] Leles S. G. Polimene L. Bruggeman J. Blackford J. Ciavatta S. Mitra A.. (2018). Modelling mixotrophic functional diversity and implications for ecosystem function. J. Plankton Res. 40, 627–642. doi: 10.1093/plankt/fby044

[ref26] Li A. Stoecker D. K. Coats D. W. (2000). Mixotrophy ingyrodinium galatheanum(DINOPHYCEAE): grazing responses to light intensity and inorganic nutrients*. J. Phycol. 36, 33–45. doi: 10.1046/j.1529-8817.2000.98076.x

[ref27] Lin C.-H. Flynn K. J. Mitra A. Glibert P. M. (2018). Simulating effects of variable stoichiometry and temperature on mixotrophy in the harmful dinoflagellate *Karlodinium veneficum*. Front. Mar. Sci. 5:320. doi: 10.3389/fmars.2018.00320

[ref28] Liu K. Chen B. Zhang S. Sato M. Shi Z. Liu H. (2019). Marine phytoplankton in subtropical coastal waters showing lower thermal sensitivity than microzooplankton. Limnol. Oceanogr. 64, 1103–1119. doi: 10.1002/lno.11101

[ref29] Liu K. Ng Y. H. Zhang S. Liu H. (2021). Effects of temperature on a mixotrophic dinoflagellate (Lepidodinium sp.) under different nutritional strategies. Mar. Ecol. Prog. Ser. 678, 37–49. doi: 10.3354/meps13865

[ref30] Lundgren V. M. Glibert P. M. Granéli E. Vidyarathna N. K. Fiori E. Ou L.. (2016). Metabolic and physiological changes in *Prymnesium* parvum when grown under, and grazing on prey of, variable nitrogen: phosphorus stoichiometry. Harmful Algae 55, 1–12. doi: 10.1016/j.hal.2016.01.002, PMID: 28073523

[ref31] Manly B. (1974). A model for certain types of selection experiments. Biometrics 30, 281–294. doi: 10.2307/2529649

[ref32] Meunier C. L. Boersma M. Wiltshire K. H. Malzahn A. M. (2016). Zooplankton eat what they need: copepod selective feeding and potential consequences for marine systems. Oikos 125, 50–58. doi: 10.1111/oik.02072

[ref33] Meunier C. L. Hantzsche F. M. Cunha-Dupont A. Ö. Haafke J. Oppermann B. Malzahn A. M.. (2012). Intraspecific selectivity, compensatory feeding and flexible homeostasis in the phagotrophic flagellate *Oxyrrhis marina*: three ways to handle food quality fluctuations. Hydrobiologia 680, 53–62. doi: 10.1007/s10750-011-0900-4

[ref34] Mitra A. Flynn K. J. Burkholder J. M. Berge T. Calbet A. Raven J. A.. (2014). The role of mixotrophic protists in the biological carbon pump. Biogeosciences 11, 995–1005. doi: 10.5194/bg-11-995-2014

[ref35] Mitra A. Flynn K. J. Tillmann U. Raven J. A. Caron D. Stoecker D. K.. (2016). Defining planktonic Protist functional groups on mechanisms for energy and nutrient acquisition: incorporation of diverse Mixotrophic strategies. Protist 167, 106–120. doi: 10.1016/j.protis.2016.01.003, PMID: 26927496

[ref36] Moeller H. V. Neubert M. G. Johnson M. D. (2019). Intraguild predation enables coexistence of competing phytoplankton in a well-mixed water column. Ecology 100:e02874. doi: 10.1002/ecy.2874, PMID: 31463931

[ref37] Montagnes D. J. Barbosa A. B. Boenigk J. Davidson K. Jürgens K. Macek M.. (2008). Selective feeding behaviour of key free-living protists: avenues for continued study. Aquat. Microb. Ecol. 53, 83–98. doi: 10.3354/ame01229

[ref38] Moorthi S. D. Ptacnik R. Sanders R. W. Fischer R. Busch M. Hillebrand H. (2017). The functional role of planktonic mixotrophs in altering seston stoichiometry. Aquat. Microb. Ecol. 79, 235–245. doi: 10.3354/ame01832

[ref39] Moreno A. R. Martiny A. C. (2018). Ecological stoichiometry of ocean plankton. Annu. Rev. Mar. Sci. 10, 43–69. doi: 10.1146/annurev-marine-121916-063126, PMID: 28853998

[ref40] Ng W. A. Liu H. Zhang S. (2017). Diel variation of grazing of the dinoflagellate *Lepidodinium* sp. and ciliate *Euplotes* sp. on algal prey: the effect of prey cell properties. J. Plankton Res. 39, 450–462. doi: 10.1093/plankt/fbx020

[ref001] R Core Team (2017). R: A language and environment for statistical computing. Vienna, Austria: R Foundation for Statistical Computing. Available at: https://www.r-project.org

[ref41] Raubenheimer D. Jones S. A. (2006). Nutritional imbalance in an extreme generalist omnivore: tolerance and recovery through complementary food selection. Anim. Behav. 71, 1253–1262. doi: 10.1016/j.anbehav.2005.07.024

[ref43] Raven J. A. (1997). Phagotrophy in phototrophs. Limnol. Oceanogr. 42, 198–205. doi: 10.4319/lo.1997.42.1.0198

[ref44] Smalley G. W. Coats D. W. (2002). Ecology of the red-tide dinoflagellate *Ceratium furca*: distribution, mixotrophy, and grazing impact on ciliate populations of Chesapeake Bay. J. Eukaryot. Microbiol. 49, 63–73. doi: 10.1111/j.1550-7408.2002.tb00343.x, PMID: 11908900

[ref45] Sterner R. W. Elser J. J. (2002). Ecological Stoichiometry: The Biology of Elements from Molecules to the Biosphere. Princeton University Press, New Jersy.

[ref46] Stoecker D. K. (1998). Conceptual models of mixotrophy in planktonic protists and some ecological and evolutionary implications. Eur. J. Protistol. 34, 281–290. doi: 10.1016/s0932-4739(98)80055-2

[ref47] Stoecker D. K. Hansen P. J. Caron D. A. Mitra A. (2017). Mixotrophy in the marine plankton. Annu. Rev. Mar. Sci. 9, 311–335. doi: 10.1146/annurev-marine-010816-060617 27483121

[ref48] Thrane J. E. Hessen D. O. Andersen T. (2017). Plasticity in algal stoichiometry: experimental evidence of a temperature-induced shift in optimal supply N: P ratio. Limnol. Oceanogr. 62, 1346–1354. doi: 10.1002/lno.10500

[ref49] Vanni M. J. (2002). Nutrient cycling by animals in freshwater ecosystems. Annu. Rev. Ecol. Syst. 33, 341–370. doi: 10.1146/annurev.ecolsys.33.010802.150519

[ref51] Wickham S. A. Wimmer R. (2019). Does Mixotrophy in ciliates compensate for poor-quality prey? Experiments with heterotrophic–mixotrophic species pairs. J. Plankton Res. 41, 583–593. doi: 10.1093/plankt/fbz052

[ref52] Wilken S. Huisman J. Naus-Wiezer S. Van Donk E. (2013). Mixotrophic organisms become more heterotrophic with rising temperature. Ecol. Lett. 16, 225–233. doi: 10.1111/ele.12033, PMID: 23173644

[ref53] Wilken S. Schuurmans J. M. Matthijs H. C. (2014). Do mixotrophs grow as photoheterotrophs? Photophysiological acclimation of the chrysophyte *Ochromonas danica* after feeding. New Phytol. 204, 882–889. doi: 10.1111/nph.12975, PMID: 25138174

