# Supplementary material for: Selective Feeding of a Mixotrophic Dinoflagellate (Lepidodinium sp.) in Response to Experimental Warming and Inorganic Nutrient Imbalance
Source: Front Microbiol. 2022 Apr 19;13:805306. doi: 10.3389/fmicb.2022.805306 (PMC9063636; doi:10.3389/fmicb.2022.805306)
Supplement: Supplementary file 1 [file Data_Sheet_1.docx]

**Supplementary Information**

**Selective feeding of a mixotrophic dinoflagellate (*Lepidodinium* sp*.*) in response to experimental warming and inorganic nutrient imbalance**

Kailin Liu, Herrick Yin-To Ng, Zuyuan Gao, Hongbin Liu

**Fig. S1** The cytograms of Becton-Dickinson FACSCalibur flow cytometer of the samples at the beginning of experiments (A) and the end of experiments (B) (C). The cytograms shows the difference in florescence signal between high-N (green, R3) and low-N (blue, R4) groups of *Rhodomonas salinia*. The X-axes is the 90° side scatter fluorescence (SSC) that can be used as a proxy of size. The Y-axes is the red auto-fluorescence emitted by Chl *a* at 680 nm (FL3).

**Fig. S2** The abundance of *Rhodomonas salina* (high N prey and low N prey) and *Lepidodinium* sp*.* at different time points (0, 2, 4, 6h) during grazing experiments under 3 different nutrient treatments (N_high_, N_Redfield_, and N_low_) at 25 °C in the first-round experiments.

**Fig. S3** The abundance of *Rhodomonas salina* (high N prey and low N prey) at 0h and 6h in control bottles (circles) and experimental bottles (triangles) during grazing experiments under 3 different nutrient treatments (N_high_, N_Redfield_, and N_low_) at three temperatures (25 °C, 28 °C, 31 °C) in the second-round experiments.


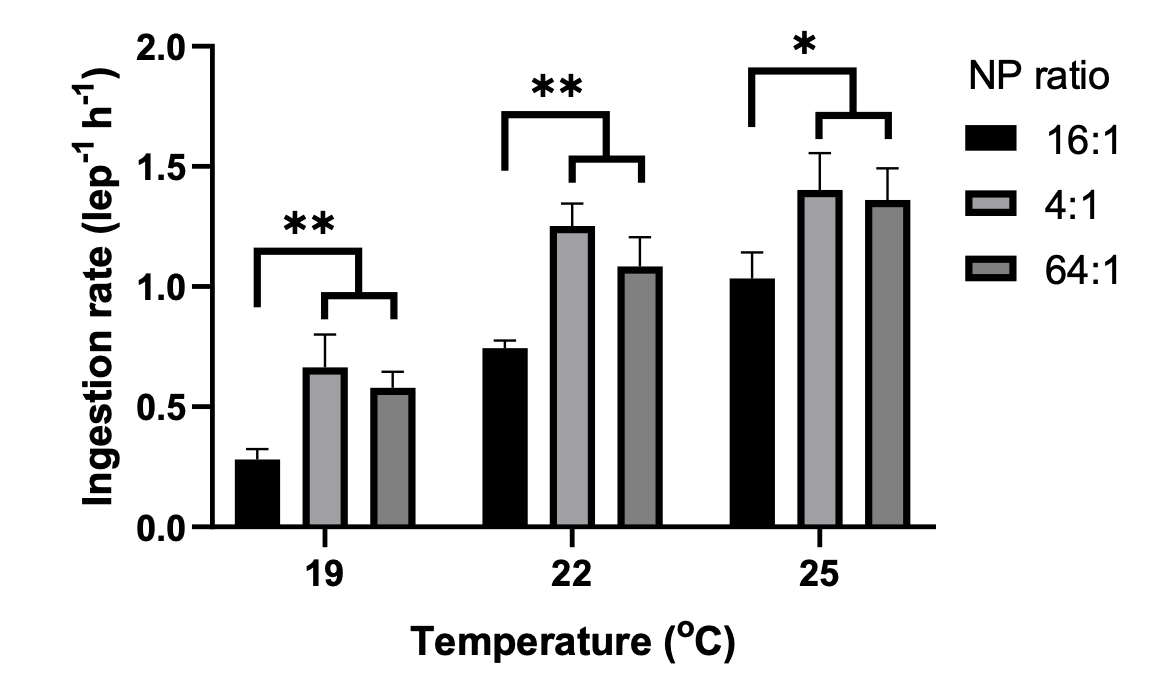


|  | **4:1** | **16:1** | **64:1** |
| --- | --- | --- | --- |
| NO_3_^-^ (μmol L^-1^) | 43.8 | 175 | 175 |
| PO_4_^3-^ (μmol L^-1^) | 7.2 | 7.2 | 1.8 |

**Fig. S4** Total ingestion rate of *Lepidodinium* sp*.* under three different nutrient treatments (N_high_, N_Redfield_, and N_low_) at three different temperatures (19, 22, and 25°C) in the second-round experiments. The asterisk indicates significant difference from N_Redfield_ conditions at each temperature (Tukey HSD Test following a one-way ANOVA; **p* < 0.05; ***p* < 0.01; ****p* < 0.001; ns: *p* > 0.05).

**Fig. S5** Ingestion rate on high-N and low-N prey and Chesson selectivity index of *Oxyrrhis marina* at 25°C under 3 different nutrient treatments (N_high_, N_Redfield_, and N_low_). Bars with the same letter(s) are not significantly different from one another.


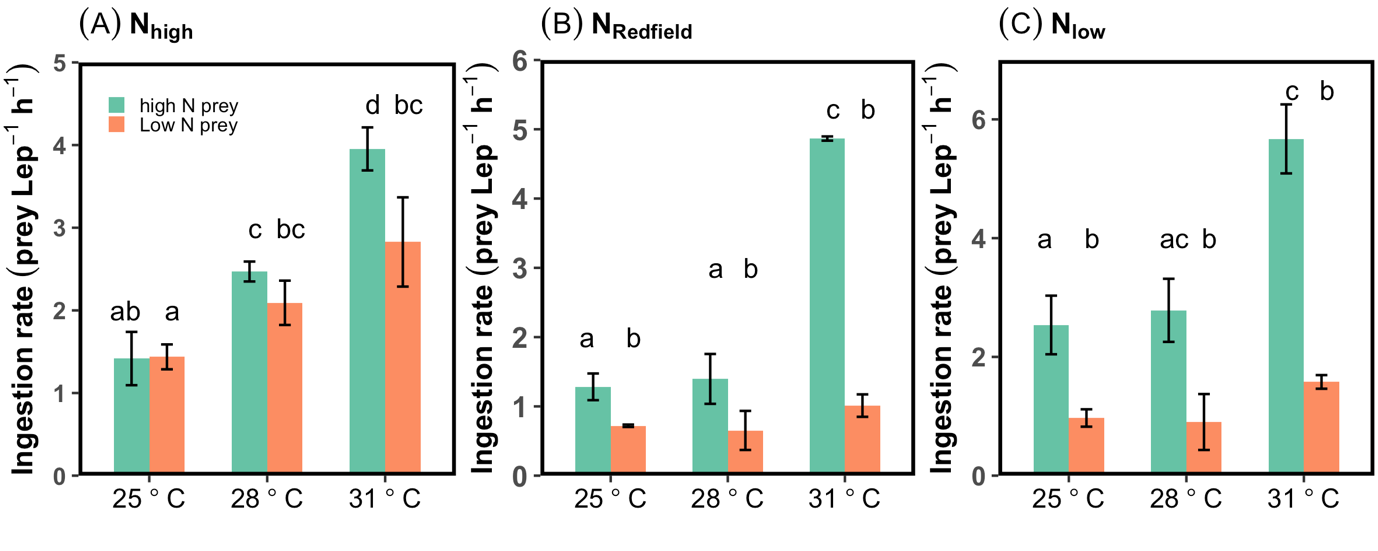


**Fig. S6** Ingestion rate of *Lepidodinium sp*. of different preys at three different temperatures under N_high_ (A), N_Redfield_ (B), and N_low_ (C) conditions. Bars with the same letter(s) are not significantly different from one another. The data was the same in Fig. 3 but regrouped by nutrient conditions focusing on the effect of temperature.
